# Baroclinic Wave Simulation Ensemble: a Machine Learning ready dataset

**DOI:** 10.1038/s41597-025-06089-z

**Published:** 2025-11-18

**Authors:** Clément Bouvier, Joona Cornér, Antti Toropainen, Andy Bowery, Glenn Carver, Sarah Sparrow, David Wallom, Victoria Anne Sinclair

**Affiliations:** 1 INAR/Physics, University of Helsinki, Helsinki, 00560 Finland; 2 e-Research Centre, University of Oxford, Oxford, OX1 3PJ United Kingdom; 3 climateprediction.net, University of Oxford, Oxford, OX1 3PJ United Kingdom

**Keywords:** Atmospheric dynamics, Scientific data

## Abstract

A large ensemble of 6,500 different baroclinic wave simulations have been run, processed and provided to study extra tropical cyclones and mid-latitudes dynamics. The data were generated using OpenIFS@home, an open science climate*prediction*.net project allowing the distribution of the computation of the ensemble with the OpenIFS 43R3v2 model. For each simulation, the cyclones were tracked and 89 features -including 16 intensity measures- were extracted. The presented dataset is composed of the raw output of the OpenIFS model for 6,388 of the 6,500 members of the ensemble and the extracted features of the tracked cyclones. The distribution of the minimum mean sea level pressure and the maximum relative vorticity at 850 hPa is plotted to enable comparison with studies that have analysed ETCs in reanalyses and climate model data. The computational failure of the missing 112 ensemble members is statistically assessed and explained. Outside of OpenIFS, the dataset and the associated code and configuration files are available and accessible.

## Background & Summary

This dataset is a collection of features extracted from an ensemble of idealised moist baroclinic wave simulations (BWS)^1,2^. Baroclinic waves are synoptic-scale, alternating low and high pressure systems that grow in the midlatitudes due to baroclinic instability. These waves are important parts of the Earth’s global circulation as they transport energy polewards. Additionally, BWS can be used to investigate the dynamics of extra tropical cyclones (ETCs).

The BWS initial background states are meteorologically and numerically stable when run without the addition of an unbalanced perturbation, and are controlled by seven entry parameters^3^. Each BWS is initialised with a different set of entry parameters sampled from a Latin Hypercube, representing a different atmospheric initial state and allowing different realistic baroclinic wave development. Then, each developing ETC is tracked and characterised with a set of 89 features. These different feature sets constitute the dataset which is presented here and is Machine Learning ready, allowing exhaustive comparisons of a vast array of different background states and the resulting baroclinic developments using traditional or deep learning methodologies. Moreover, this dataset can be used with standard meteorological methods and as an alternative to re-analysis datasets to study ETCs. This comparison can be extended to understand the intricate relationship between the background atmospheric state and the eventual intensity of a cyclone that develops in it.

The dataset consists of one xml file and two repositories. The first repository contains 6,388 folders, each representing one BWS as simulated by the Open Integrated Forecasting System (OpenIFS) 43R3v2^4^. The raw outputs are compressed and stored in these folders. In total 28 variables are output on either surface levels or on 28 pressure levels at a horizontal resolution of 125 km at the equator and 3 hour temporal resolution. The second repository has 22,259 comma-separated value files (.csv) containing 89 features extracted for each BWS and associated ETC. The features can be classified into four categories: background-related features, track-related features, dynamical intensity measures, and impact-relevant intensity measures.

## Methods

This section describes the methods and infrastructures used to produce the dataset, including the toolboxes used and Application Programming Interface (API).

### Moist baroclinic wave simulation

General Circulation Models (GCMs) are the cornerstone of weather forecasting and climate simulation^5^. They can be used to produce operational weather forecasts, predict future climate^6^, but also to simulate idealised weather systems enabling specific phenomena, such as convection^7^ or baroclinic waves^8^ to be studied. Baroclinic waves are synoptic weather phenomena of high and low pressure systems that develop in the mid-latitudes. These waves are fundamental to understand Earth’s global circulation as they transport energy and moisture polewards^2,9,10^. To study these phenomena, moist baroclinic wave simulations are performed using the Open Integrated Forecasting System (OpenIFS) cycle 43R3v2^4^.

OpenIFS is a version of the Integrated Forecasting System of the European Centre for Medium-Range Weather Forecasts (ECMWF). Cycle 43R3 of IFS was operational at ECMWF from July 2017 to June 2018^4^. The initial background state for these simulations is expressed analytically and setup through the configuration files in an aquaplanet setting. As a result, the jet structure and strength, the average virtual temperature, the surface relative humidity, the lapse rate and the surface roughness can be easily modified. In total, seven input parameters can be controlled to produce different initial background states, and subsequently different baroclinic wave developments. Previous work^3^ can be consulted for more details on the background state, the baroclinic wave development and the implementation in OpenIFS 43R3v2. Each baroclinic wave simulation is run with a spatial resolution of T_*L*_159, which corresponds to a specific spectral truncation used to compute the linear terms in the equations of the model^11^. The final grid cell size is approximately 125 km at the equator with 91 sigma levels, and the simulations are global. Alongside the spatial resolution, a model time step of 900 s (15 min), an output frequency of 3 h and a length of simulation of 20 days are chosen. The following physical parameterization schemes have been activated: vertical diffusion (LEVDIF), surface processes (LESURF), large-scale condensation (LECOND), mass-flux convection (LECUMF), prognostic cloud scheme (LEPCLD) and evaporation of precipitation (LEEVAP). The text in parenthesis refers to the OpenIFS namelist option that is set to true to activate each physical parameterization scheme. In addition, the negative humidity fixer (LEQNGT) is also turned on^12^. The radiation schemes have not been triggered. Depending on the initial background state, multiple low and high pressure systems develop. Some initial conditions lead to no development at all. All code and configuration files related to the moist baroclinic wave simulations can be found on this Zenodo repository^13^.

### Ensemble generation

To generate the ensemble, the seven input parameters are used to define a 7-dimensional hypercube. The input parameters, along with their maximum and minimum values are given in Table 1. A Latin Hypercube Sampling (LHS)^14^ is performed in this parameter space to define a list of 6,500 different configurations. The code used to generate the LHS can be found in a GitLab repository^15^. Each configuration results in a specific set of initial conditions and thus a unique baroclinic wave simulation, which was simulated using OpenIFS@home^16^. OpenIFS@home is an open science climate*prediction*.net (CPDN)^17^ project allowing the fast computation of the computationally heavy ensemble of OpenIFS forecasts using the Berkeley Open Infrastructure for Network Computing (BOINC)^18^ framework. Five days after the start of the ensemble computation, 80% of the members were returned and transferred to the CSC - IT centre for science Ltd^19^ infrastructure for further processing as detailed in the next section.

**Table 1:** Details of the 7 input parameters detailed in the Methods section. Each line correspond to one of the seven input parameter.

Input parameter	Units	Min	Max	Data type
n, Jet width	Dimensionless	1	6	integer
b, Jet height	Dimensionless	0.5	2.5	float
*u*_0_, Average zonal wind speed	m s^−1^	15.0	75.0	float
*T*_*v*,0_, Average virtual temperature	K	265.0	300.0	float
*RH*_0_, Surface level relative humidity	%	0.0	80.0	float
Lapse rate	K km^−1^	0.003	0.006	float
Charnock parameter	Dimensionless	0.01	0.035	float

### Ensemble processing

**Fig. 1:**
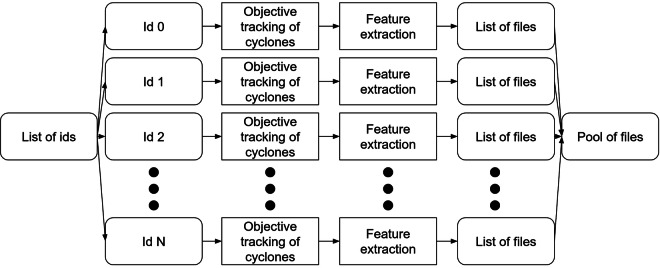
Distributed workflow for objective cyclone tracking and feature extraction.

All the BWS are processed in order to 1) track the ETCs which develop in the simulations and 2) extract features which further characterise the background state and the developing cyclones. The workflow is presented in Fig. 1 and is fully implemented using the HyperQueue API^20^ on the CSC infrastructure^19^. From a list of identification tags (the experiment IDs used by OpenIFS), each BWS is processed independently, between 1 and 4 files are generated and pooled together. The next sections describe in detail the objective cyclone tracking and the feature extraction process.

#### Objective cyclone tracking

Cyclone tracks are identified with the objective feature tracking software TRACK^21–23^. TRACK uses a Lagrangian approach of tracking individual cyclones by identifying extrema in a given field and following them through time. In order to track developing cyclones, the relative vorticity at 850 hPa (VO-850) at the T_*L*_159 resolution is first truncated to the T42 spectral resolution (310 km at the equator) and the planetary scale waves (wavenumbers 1-5) are excluded. This truncation ensures that very large- and small-scale features are excluded and only synoptic-scale cyclones are identified. TRACK produces output which consists of the horizontal location (longitude and latitude) and magnitude of the T42 VO-850 maxima for each time frame in each cyclone track. Then, the maximum relative vorticity within 2° geodesic radius is localised using relative vorticity at 700 hPa, 600 hPa, 500 hPa, 400 hPa, 300 hPa and 200 hPa in order to compute the tilt of the cyclone at these different pressure levels with VO-850 as the reference. The tilt is computed iteratively starting from the tracked relative vorticity at 850 hPa. Using the T42 maxima at the next pressure levels (700 hPa), the steepest ascent maximisation within a 5° geodesic radius is estimated using B-spline and the tilt is computed^24^. The tilt is computed alongside the objective tracking^25^. Finally, the cyclone tracks based in VO-850 are filtered to exclude stationary, weak and short-lived systems. Therefore, the tracks need to have a T42 VO of at least 1 × 10^−5^ s^−1^, be at least 1000 km long, and last for at least two days. All TRACK’s configuration files can be found in a GitLab repository^15^. The first three cyclones objectively identified in each BWS simulation by TRACK are kept for the feature extraction process.

#### Feature extraction

**Fig. 2:**
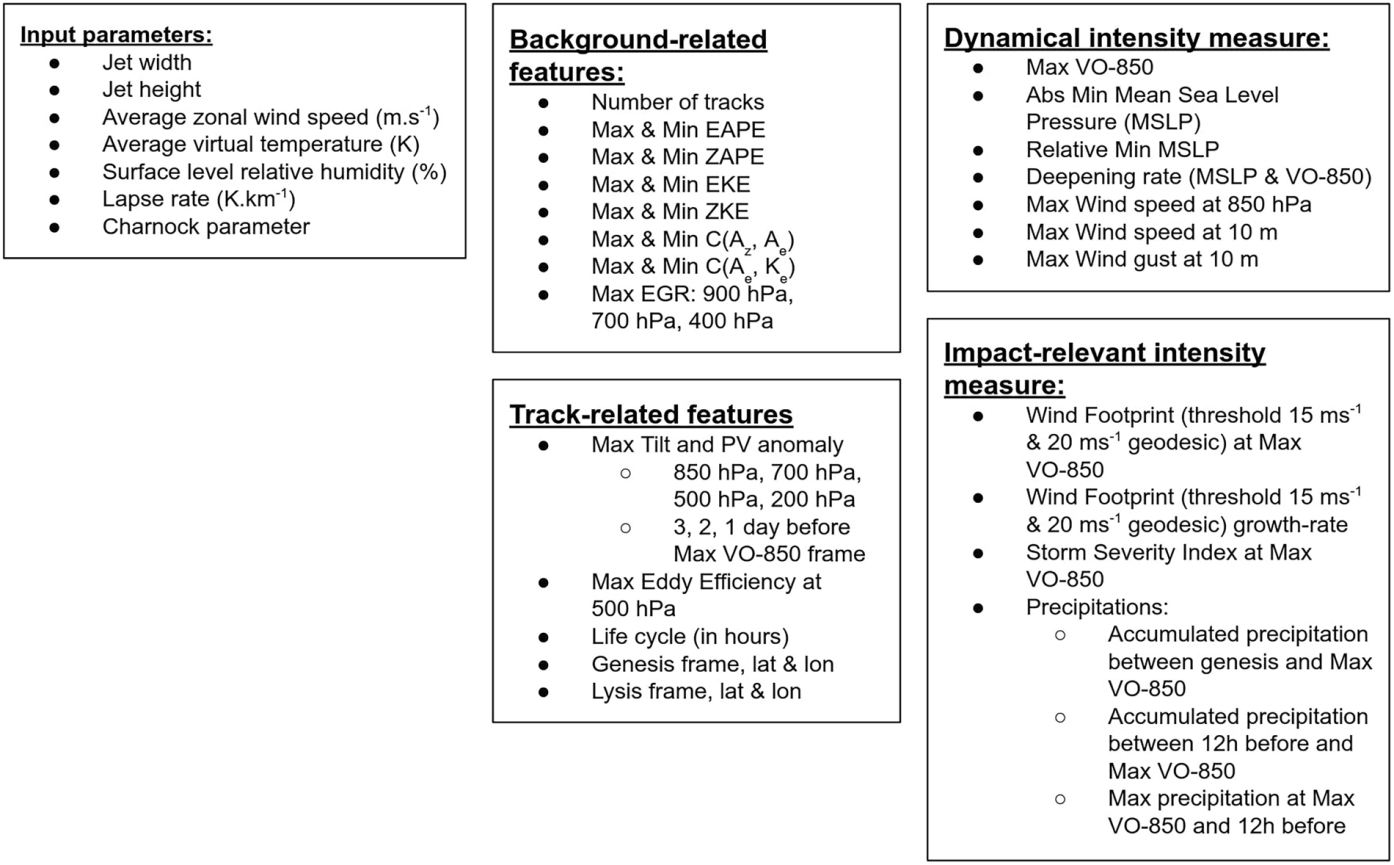
Taxonomy of the extracted features. The time frame of occurrence of each represented feature is also extracted if not computed at Max VO-850 time.

The feature extraction process can be decomposed into two parts and the features into four categories as represented in Fig. 2. The processing pipeline has been developed using Python.

First, background-related features are extracted. These features are only dependent on the background state and its evolution, meaning that these features are extracted regardless of the development of baroclinic waves. For each BWS, the time-series of the four energies of the Lorenz energy cycle are computed^26,27^: the Zonal mean Available Potential Energy (ZAPE), the Eddy Available Potential Energy (EAPE), the Eddy Kinetic Energy (EKE), and the Zonal mean Kinetic Energy (ZKE). The time-series of conversion terms between the ZAPE and EAPE, and between EAPE and EKE are averaged and extracted between 30° N and 60° N. The details of this calculations are shown in Toropainen (2024)^28^. The absolute maximum, minimum, and their respective time frame of occurrence are extracted. Finally, the maximum zonal mean Eady Growth Rate (EGR) values between 30° N and 60° N at each pressure levels considered (900, 700, 400 hPa) are computed from the potential temperature at a pressure level above and below^29^.

Then, the track-related features and the intensity measures are calculated. These features are extracted if at least 1 track is detected during the objective cyclone tracking for a given BWS. From the tracking process, the time frames of Genesis and Lysis are the first and last frame of the track respectively. The life cycle duration is the number of frames corresponding to the difference between Genesis and Lysis. The PV anomaly is computed as the difference between the PV field and the PV zonal average at every time frame. Then, the average value within a 2° geodesic radius from the potential vorticity maximum is computed^30,31^. The tilt and PV anomalies are extracted 3 days, 2 days, and 1 day before the frame of maximum tracked VO-850 at all pressure levels considered. The maximum Eddy Efficiency^32^ is computed as the maximum of the Eddy Efficiency averaged over a 10° geodesic radius centred on the cyclone’s center at 500 hPa.

All intensity measures are computed according to the study by Cornér *et al*.^33^ and using the code available in a GitLab repository^15^. A total of 16 intensity measures are computed including maximum vorticity, wind speed (850 hPa, 10 m), wind gust (10 m), the minimum mean sea level pressure (MSLP) and its growth-rate, the wind footprint with 15.0 and 20.0 m s^−1^ threshold and their respective growth-rates, and a Storm Severity Index (SSI)^34^. Finally, three precipitation measures are computed, two accumulated total precipitation (between genesis and the maximum vorticity frame, and the 12 hours before the maximum vorticity frame), and one instantaneous precipitation measure 12 hours before the maximum vorticity frame^35^.

## Data Records

The dataset is available in FairData.fi^36^. The repository containing the data consists of three parts which are accessible independently. First, the xml used to submit the batch of jobs to the OpenIFS@home infrastructure. Each case is described by a *unique_member_id* and a set of *parameters*. The *unique_member_id* is a set of four walpha-numerical characters unique to a set of *parameters*, it is assigned during the generation of the ensemble and ranges from *a000* to *a50j*. The set of *parameters* corresponds to the seven input parameters used to construct the background state of the baroclinic wave simulations and are presented in Table 1^3^. The parameters are randomly selected and thus there is no meaningful relationship between the *unique_member_id* and the *parameters*.

Secondly, a folder named *batch_1018* which contains the raw output of the OpenIFS@home infrastructure. Each sub-folder corresponds to one of the successful runs (6,388 sub-folder in total, see the Technical Validation section). The *unique_member_id* is included in the name of the sub-folder, which contain a collection of 20 zip archives in which the raw gridded OpenIFS output (GRIB files) is stored. The total size of this dataset is 10.34 TB. A comprehensive description of the *batch_1018* folder is given Table 2.

**Table 2:** Data breakdown for each baroclinic wave simulation.

Number of BWS:	6,388
Data size per BWS:	3.9 GB
Number of compressed files per BWS:	20
Number of uncompressed files per BWS:	485
Spatial resolution:	T_*L*_159 L91
Output frequency and length of simulation:	3 h and 20 days
List of outputted OpenIFS pressure levels (19 levels):	1000 hPa, 950 hPa, 900 hPa, 850 hPa, 800 hPa,
	750 hPa, 700 hPa, 650 hPa, 600 hPa, 550 hPa,
	500 hPa, 450 hPa, 400 hPa, 350 hPa, 300 hPa,
	250 hPa, 200 hPa, 150 hPa, 100 hPa
Number of OpenIFS parameters:	28
List of 3D OpenIFS fields:	Potential vorticity
	Geopotential
	Temperature
	Zonal wind (U)
	Meridional wind (V)
	Specific humidity
	Vertical velocity (W)
	Relative vorticity
	Divergence
	Relative humidity
	Specific cloud liquid water content
	Specific cloud ice water content
	Fraction of cloud cover
List of 2D OpenIFS fields:	Surface pressure
	Logarithm surface pressure
	Sea surface temperature
	Maximum 10 metre wind gust since previous post-processing
	Convective available potential energy
	Maximum 10 metre wind gust in the last 6 hours
	Total column vertically-integrated water vapour
	Large-scale precipitation
	Convective precipitation
	Mean sea level pressure
	10 metre U wind component
	10 metre V wind component
	2 metre temperature
	Evaporation
	Forecast surface roughness
Total size of this repository (compressed raw data):	10.34 TB

Lastly, a folder named *ExtractedFeatures* contains the collection of features computed (see Fig. 2) as described in the previous section. There are 22,259 files, each respecting the following nomenclature: *unique_member_id*_*general* for the background-related features, *unique_member_id*_*0/1/2* for the track-related features and the intensity measures respectively for the first, second and third baroclinic wave developing in the *unique_member_id* case. A comprehensive description of the *ExtractedFeatures* folder is given in Table 3.

**Table 3:** Data breakdown for the feature extraction.

Number of Features:	89
Number of BWS with 0 baroclinic development:	372
Number of BWS with 1 cyclone:	305
Number of BWS with 2 cyclones:	95
Number of BWS with 3 or more cyclones:	5,248
Total number of files:	22,259
Total size of the repository:	39.3 MB

## Technical Validation

Of the 6,500 members of the ensemble, 6,388 members have been processed successfully. Of the successful runs 80% have been processed within 5 days of the ensemble being launched on the OpenIFS@home infrastructure^16^. The remaining 20% of the successful runs have been returned within one month of launching the ensemble. OpenIFS@home is an open science and distributed infrastructure meaning that it is dependent on a higher number of technical and human parameters than a traditional High-Performance Computing (HPC) setup, which explains the late return of some of the successful cases. The remaining 112 cases have not been returned (12) or have failed (100). These failures are caused by unrealistically strong cyclones developing which resulted in exceptionally strong updraft winds, causing the OpenIFS 43R3v2 model to become numerically unstable and crash. The 112 unsuccessful runs will be called “hard fail runs” in the rest of the manuscript.

**Fig. 3:**
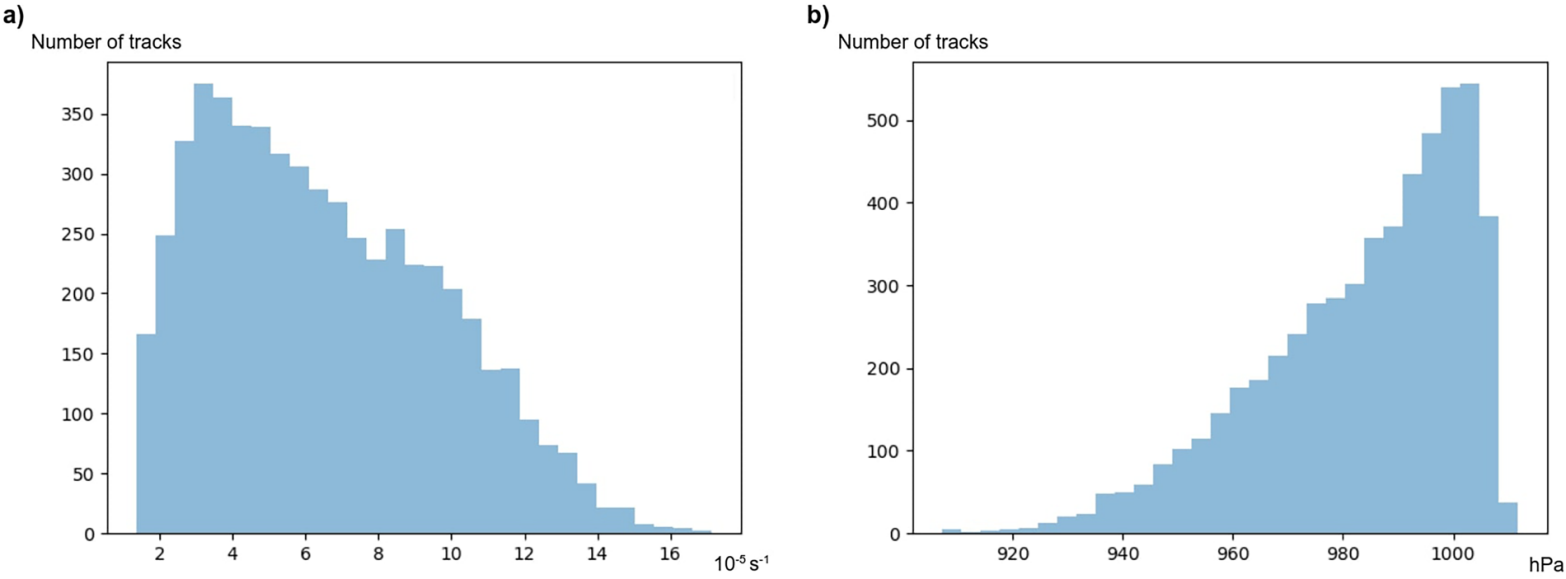
Total distribution of (**a**) the maximum VO-850 and (**b**) the minimum MSLP. Note that the y-axes differ between the two panels.

**Fig. 4:**
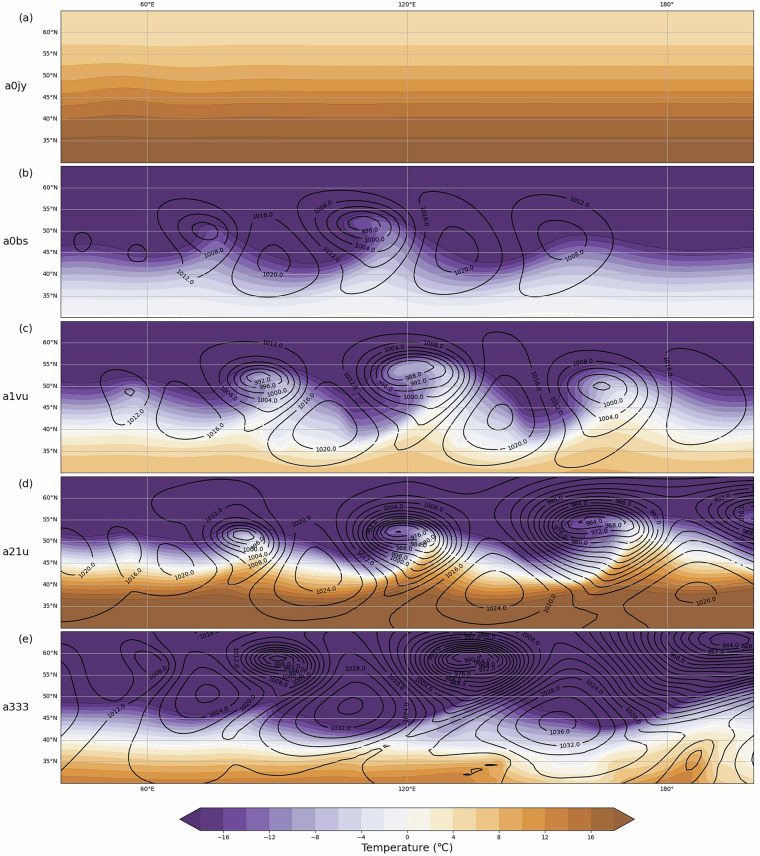
Baroclinic development for 5 cases at t = 204 hour, from weak to strong waves. The black contours show mean sea level pressure (hPa), and the shading shows the temperature (°C) at 850 hPa. The label on the left correspond to the member ID of the case, their input parameters are given Table 5. Note: this figure does not show the whole (global) model domain; the x axis ranges from 40–220° E, while the y axis ranges from 30–65° N.

To test the validity of the simulated ETCs, the distribution of the maximum VO-850 and minimum MSLP for the first tracked cyclone in all 6388 successfully processed simulations is plotted in Fig. 3. The two distributions are skewed towards the most intense values, which is similar to the distributions for ETCs found in previous studies which analysed ETCs in the historical climate using reanalysis datasets and in the future climate using climate model simulations^25,33,37–39^. The baroclinic wave simulations^3^ therefore result in ETCs with reasonable intensity measure distributions compared to current or projected future climates. Furthermore, a visual inspection of relevant meteorological variables plotted on a map reveal features, such as cold fronts and warm sectors, which resemble those found in analyses from satellite images, reanalyses or model output. Hence we conclude that the ETCs in these idealised simulations resemble ETCs found in the real world. However, the set of input parameters strongly influence the speed and strength of the ETCs development as shown in Table 4 and Fig. 4 for five arbitrarily chosen cases. After 8.5 days of the development (the time step shown in Fig. 4) different combinations of input parameters have resulted in a varying number of mature low pressure systems of varying intensities. Cases with more and deeper low pressure systems undergo more rapid and intense development than those with fewer and shallower ones. For example, from a narrow, low and weak jet in Fig. 4a there is no baroclinic wave development at all. The meridional temperature gradient in this case is weak indicating little baroclinicity which results in slow development of baroclinic waves, as theoretically expected from the Eady model^40^. In another case with a wide, high and strong jet, and thus large baroclinicity, (Fig. 4e) many deep ETCs associated with large pressure gradients and frontal features can be seen. As a result, the dataset is considered of interest for the study of current and alternative climates. Future work will include comparison with CMIP6^6^ projections and in-depth comparison with ERA5^33,41^ tracked cyclones.

**Table 4:** Experiment IDs and input parameters for the 5 cases presented Fig. 5. Number are truncated at 2 significant digits.

Member ID	n	b	*T*_*v*,0_	*u*_0_	*RH*_0_	Laps rate	Charnock parameter
a0jy	6	0.91	297.08	21.04	44.39	0.0052	0.030
a0bs	4	1.57	269.60	42.17	3.14	0.0044	0.027
a1vu	4	1.69	279.77	42.82	55.59	0.0057	0.026
a21u	3	2.025	292.36	68.00	70.79	0.0051	0.032
a333	1	2.31	279.68	56.86	79.94	0.0059	0.019

**Fig. 5:**
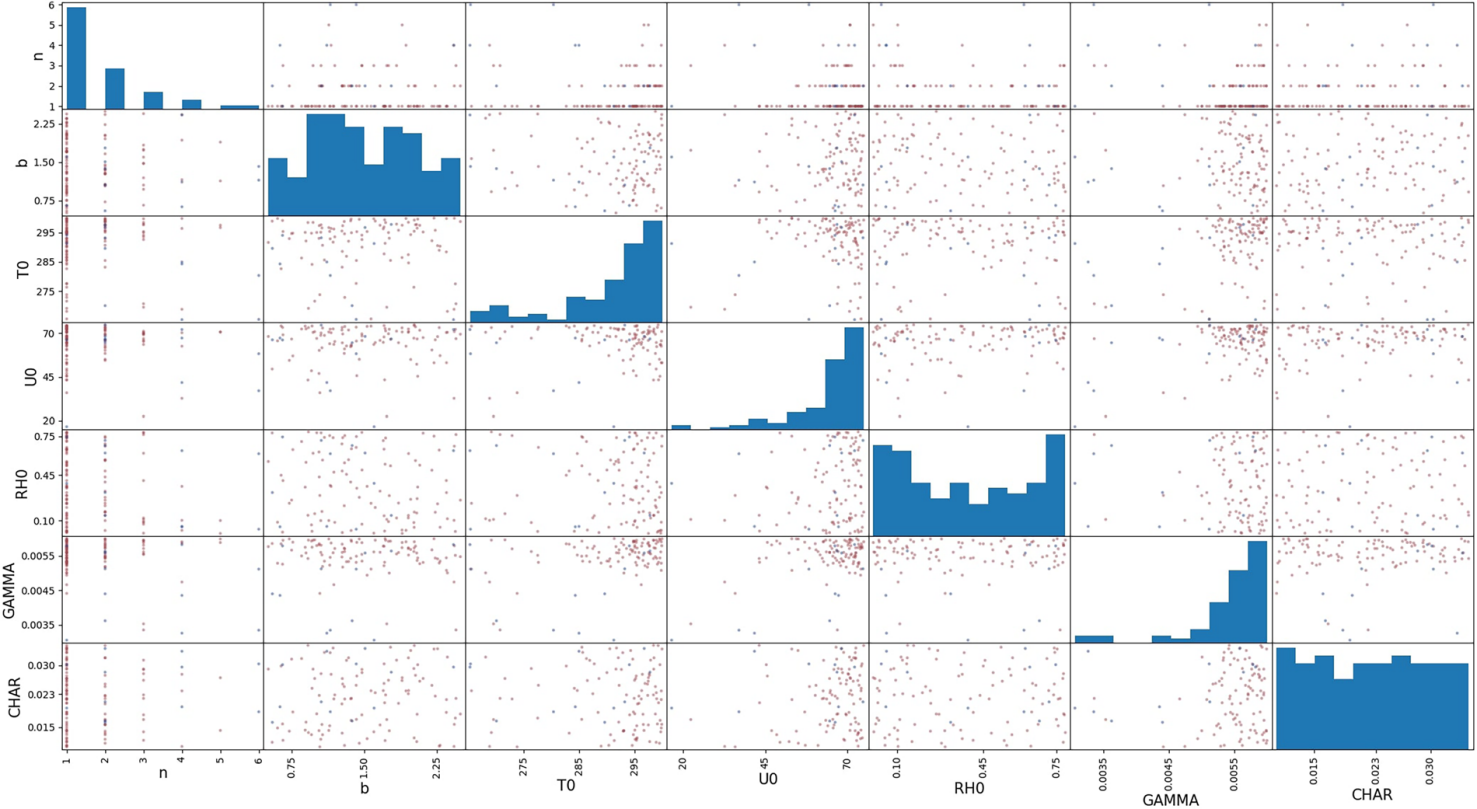
Projection of missing and hard fail runs in the initial hypercube, brown dots represent hard fail runs, cyan dots the missing runs. The diagonal represents the missing and hard fail distributions of the corresponding input parameter.

Figure 5 presents a projection of the missing and hard fail run (112 runs total) in the 7-dimensions hypercube. On the diagonal, the total distribution of the runs is represented. These distributions are compared to the original uniform distributions in order to assess the dependency of the missing or hard fails runs on the seven entry features (see Table 1) with the Mann-Whitney U-test and the Cramér-von Mises test. To consider that an entry feature is increasing the probability of a missing or hard fail run, both tests have to have a p-value below the confidence level. The confidence level is set at 5% for both tests. The results of the statistical tests are presented in Table 5. The missing run distributions depend on *u*_0_, but due to the low sample size for the missing runs, no conclusion can be reasonably drawn for these 12 cases. Concurrently, n, *T*_*v*,0_, *u*_0_ and the lapse rate values (see Table 1) increase the probability to have a hard fail run. The hard fail runs are due to the unrealistic background states which can be generated by our implementation^3^. High lapse rate (greater than 0.005 K km^−1^), initial virtual temperature (superior to 295 K), wind speed (superior to 60 m s^−1^), with a wide jet stream (small n) create extreme initial conditions, making the OpenIFS 43R3v2 model to become numerically unstable and to crash.

**Table 5:** Statistical tests for missing and hard fail runs, p-value are written if both tests are below the level of confidence (*α* = 5%).

Statistical test	n	b	*T*_*v*,0_	*u*_0_	*RH*_0_	Laps rate	Charnock parameter
Missing run: U-test p-value	—	—	—	0.014	—	—	—
Missing run: Cramer p-value	—	—	—	9.98e^−3^	—	—	—
Hard fail run: U-test p-value	1.22e^−28^	—	4.92e^−19^	8.09e^−32^	—	7.77e^−33^	—
Hard fail run: Cramer p-value	1.9e^−7^	—	9.50e^−11^	9.69e^−10^	—	2.73e^−9^	—

## Usage Notes

To manipulate the outputted GRIB files by OpenIFS@home in the folder *batch_1018*, python scripts can be found in a Zenodo repository in the *plotting_scripts* folder^13^. The script *Usage_Script.py* uses the xml file and the *ExtractedFeatures* folder to produce the Figs. 3 and 5, and the statistical tests presented in Table 5. By modifying the beginning of the script, each extracted feature can be filtered and / or plotted. The *Usage_Script.py* is available in a GitLab repository^15^.

## Data Availability

The modified subroutines of OpenIFS, a standalone version to compute the initial zonally uniform fields for wind, the temperature and the geopotential as detailed in Bouvier *et al*. ^3^ are available in a Zenodo repository^13^. The entire dataset is available in FairData.fi^36^. The dataset can be access *via* the Metax API^42^.

## Code availability

The licence for using the OpenIFS CY43R3 model can be requested from ECMWF user support (openifs-support@ecmwf.int) and is given free of charge to any academic or research institute. The modified subroutines of OpenIFS, a standalone version to compute the initial zonally uniform fields for wind, the temperature and the geopotential as detailed in Bouvier *et al*. ^3^ are available in a Zenodo repository^13^. OpenIFS43r3 with the specific changes required to reproduce this work is available on the CPDN platform as OpenIFS@Home, access to which can be obtained via^43^. A GitLab repository consists of the namelists for TRACK^21–23^, the feature extractor code to produce the *ExtractedFeatures* folder, the Latin Hypercube Sampling code, and the *Usage_Script.py*^15^. The entire dataset is available in FairData.fi^36^. The dataset can be access *via* the Metax API^42^.
